# Chromatin conformation and histone modification profiling across human kidney anatomic regions

**DOI:** 10.1038/s41597-024-03648-8

**Published:** 2024-07-18

**Authors:** Haikuo Li, Dian Li, Benjamin D. Humphreys

**Affiliations:** 1 Division of Nephrology, Department of Medicine, Washington University in St. Louis, St. Louis, MO USA; 2 Department of Developmental Biology, Washington University in St. Louis, St. Louis, MO USA

**Keywords:** Epigenetics, Genetics, DNA methylation

## Abstract

The three major anatomic regions of the human kidney include the cortex, medulla and papilla, with different functions and vulnerabilities to kidney diseases. Epigenetic mechanisms underlying these anatomic structures are incompletely understood. Here, we performed chromatin conformation capture with Hi-C and histone modification H3K4me3/H3K27me3 Cleavage Under Targets and Release Using Nuclease (CUT&RUN) sequencing on the kidney cortex, medulla and papilla dissected from one individual donor. Nuclear suspensions were generated from each region and split subjected to paired Hi-C and CUT&RUN sequencing. We evaluated the quality of next-generation sequencing data, Hi-C chromatin contact matrices and CUT&RUN peak calling. H3K4me3 and H3K27me3 histone modifications represent active and repressive gene transcription, respectively, and differences in chromatin conformation between kidney regions can be analyzed with this dataset. All raw and processed data files are publicly available, allowing researchers to survey the epigenetic landscape across regional human kidney anatomy.

## Background & Summary

The human kidneys play an essential role in waste removal, fluid balance, blood pressure regulation and multiple endocrine functions. Chronic kidney disease is characterized by kidney function decline and affects over 800 million individuals worldwide^1–4^. The cortex, medulla and papilla are three major anatomic structures of the human kidney. The three regions contain different populations of kidney cell types and present distinct vulnerability to various kidney diseases. For example, proximal tubular cell, predominantly present in the kidney cortex, is the major target of diabetic kidney disease and drug responses^5–8^. The kidney medulla and papilla, on the other hand, are more susceptible to cystic kidney disease, autosomal-dominant tubulointerstitial kidney disease and renal hypodysplasia^9^. Therefore, understanding the multiomic signatures of these different kidney anatomic regions is crucial to identify new therapeutic targets.

Gene expression in mammalian cells is precisely controlled by epigenetic mechanisms such as chromatin modifications and regulation of 3-dimensional (3D) chromatin architectures^10–15^. We recently profiled transcriptomics, open chromatin accessibility and metabolomics profiles from cells of cortex, medulla and papilla in human kidneys^16,17^, but other epigenomic modalities, including chromatin conformation^18^ and histone modification^19^ profiles, across these kidney anatomic regions, remain unexplored. Although the 3D chromatin architecture or histone modifications such as H3K4me3 and H3K27Ac have been profiled in primary human kidney tissues^20–24^, these studies only included the cortex in their sample cohorts. A recent study successfully performed Hi-C (a technology of chromatin conformation capture) on human kidney cortex and medulla samples^25^, but histone modification profiling was still lacking.

**Fig. 1:**
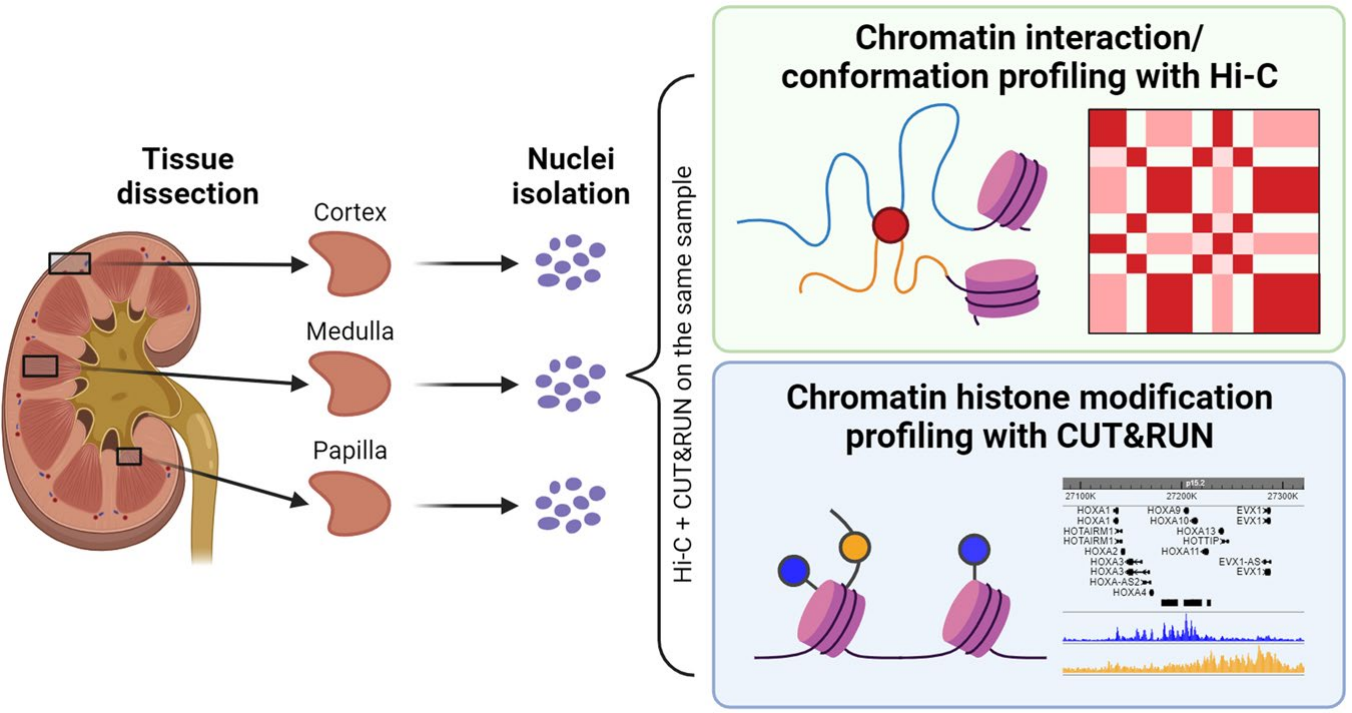
Workflow schematic. Kidney cortex, medulla and papilla tissues were obtained from a healthy donor. Nuclei were extracted from these tissues and processed for two epigenomic profiling assays, Hi-C and CUT&RUN, in parallel.

A major challenge in profiling both chromatin conformation and histone modifications from the same sample is that sequencing each modality requires a large number of cells or nuclei as input in library preparation. Here, we leveraged our recently described high-yield nuclei extraction method^26,27^ and performed Hi-C and Cleavage Under Targets and Release Using Nuclease (CUT&RUN) sequencing of histone modifications (H3K4me3 and H3K27me3) in parallel on the kidney cortex, medulla and papilla samples dissected from one individual donor (Fig. 1). This dataset of simultaneous profiling of chromatin conformation and histone modifications is publicly available^28,29^, allowing investigation of the epigenetic landscape across regional human kidney anatomy.

## Methods

### Donor description

This research complies with all relevant ethical regulations and has been approved by the Washington University Institutional Review Board. Human kidney cortex, medulla and papilla tissue samples were obtained from the Washington University Kidney Translational Research Core Biobank with consent managed by the core. The kidney sample was discarded for transplantation and was from a deceased organ donor (67-year-old female). These studies were approved by the Washington University in St. Louis Institutional Review Board, protocol #201601020. This approval included consent to publish genomic data. The donor has a normal kidney function, with creatinine level at sampling as 0.6 mg/dL and with mild interstitial fibrosis. All tissues samples were frozen with liquid nitrogen and stored at −80 °C before use. To be noted, the aim of this study is to provide a high-quality paired Hi-C and CUT&RUN sequencing dataset on a healthy human control, instead of including a large cohort of samples to generate new biological hypotheses.

### Nuclear suspension generation

Generation of nuclear suspension from frozen kidney samples was performed as previously described with minor modifications^26,27^. Briefly, Nuclei EZ Lysis Buffer (NUC101, Sigma) was supplemented with EDTA-free protease inhibitor tablets (5892791001, Roche). Tissues were exposed to the ice-cold lysis buffer, minced with a razor blade and homogenized with Dounce Tissue Grinders (885303-0002, Kimble) with the large pestle. The homogenate was filtered through a 200-µm Mini Strainer (43-10200-60, pluriSelect) and cell homogenization was performed with the Dounce Tissue Grinder again with the small pestle. The homogenate was incubated in the buffer for 3 minutes and then filtered through a 40-µm Mini Strainer (43-10040-60, pluriSelect). The homogenate was centrifuged at 500 × g for 4 minutes at 4 °C and the pellet was resuspended with the lysis buffer. After 5-minute incubation, the suspension was centrifuged at 500 × g for 4 minutes at 4 °C and the nuclei pellet was resuspended with 2 mL nuclei buffer (10 mM Tris-HCl pH 7.5, 10 mM NaCl, 3 mM MgCl_2_) supplemented with 3% BSA (B9000S, NEB) by pipetting 10 times. The concentration of nuclear suspension was counted with a Countess Automated Cell Counter.

### Hi-C library generation

Hi-C was performed with the Arima-HiC kit (A510008, Arima Genomics) following the manufacture’s manual with minor changes. Briefly, we transferred 5–7 million fresh nuclei and reconstituted the volume to 5 mL with nuclei buffer (10 mM Tris-HCl pH 7.5, 10 mM NaCl, 3 mM MgCl_2_) supplemented with 3% BSA (B9000S, NEB). Then, crosslinking was performed according to the manufacture’s protocol (“Crosslinking – Standard Input” section in A160134 v01) by adding 286 µL 37% formaldehyde (final concentration 2%). The BSA-supplemented nuclei buffer was consistently used during nuclei washes. Nuclei were aliquoted with 1 million nuclei per aliquot and stored at −80 °C for subsequent reactions. Each aliquot containing 1 million nuclei is considered as a replicate and we included two replicates for each kidney anatomic region in this study.

**Fig. 2:**
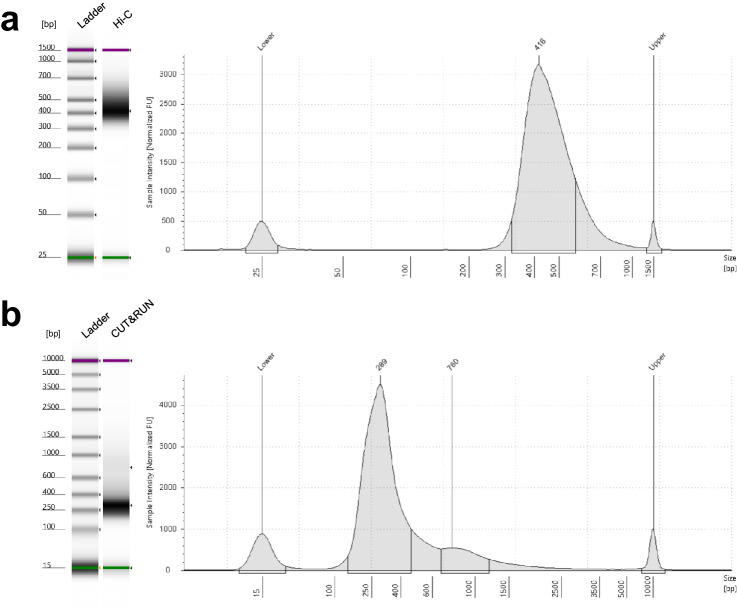
TapeStation profiles of representative Hi-C and CUT&RUN libraries. (**a**) TapeStation HSD1000 gel image of the ladder and a Hi-C library (left) and the electropherogram trace of the Hi-C library (right). (**b**) TapeStation HSD5000 gel image of the ladder and an H3K4me3 CUT&RUN library (left) and the electropherogram trace of the CUT&RUN library (right).

The 1-million nuclei pellet was resuspended with 25 µL nuclease-free water and 20 µL was proceeded with the manufacture’s protocol (“Arima-HiC Protocol” section in A160134 v01) for restriction enzyme digestion, end filling, ligation and purification. Hi-C library preparation was performed with the Arima Library Prep Kit v2 (A303011, Arima Genomics) with minor changes. Briefly, DNA fragmentation was performed with a Diagenode Bioruptor Pico device (6-7 cycles of 15 seconds’ on and 90 seconds’ off on 100 µL DNA products). DNA size selection, biotin enrichment and library amplification were performed according to the manufacture’s protocol (A160432 v02). A total of 8 cycles were used for library amplification and the final library was eluted with 25 µL nuclease-free water. The final libraries were visualized and quantified with a TapeStation instrument, with a representative Hi-C library trace shown in Fig. 2a. The i7 and i5 index sequences of each library are presented in Table 1.

**Table 1:** Index sequences of 6 Hi-C libraries presented in this study.

Region	Replicate	i7	i5
Cortex	Replicate1	AGTCGCGA	TAGACCAA
	Replicate2	CGGTAGAG	AGTCGCGA
Medulla	Replicate1	TCAGCATC	AAGGAGCG
	Replicate2	AGAAGCAA	TCAGCATC
Papilla	Replicate1	GCAGGTTC	AGAAGCAA
	Replicate2	AAGTGTCT	GCAGGTTC

### CUT&RUN library generation

CUT&RUN was performed with the CUTANA™ ChIC / CUT&RUN Kit v3 (Cat# 14-1048, EpiCypher) with minor modifications. Briefly, after counting the concentration of the nuclear suspension, the suspension was spined down at 600 × g for 4 minutes at 4 °C and reconstituted to 5 million/mL with nuclei buffer (10 mM Tris-HCl pH 7.5, 10 mM NaCl, 3 mM MgCl_2_). Then, 100 µL suspension containing 500,000 nuclei was mixed with 10 µL activated EpiCypher ConA Beads. Each 100 µL aliquot containing 500,000 nuclei is considered as a replicate and we included two replicates for each reaction in this study.

For antibody binding, 0.5 µg H3K4me3 (Cat # 13-0041k, EpiCypher) and H3K27me3 (Cat # 13-0055, EpiCypher) antibodies were used and 0.5 µg rabbit IgG antibody (Cat # 13-0042k, EpiCypher) was used as a negative control. As recommended by the manufacture’s manual, a SNAP-CUTANA™ K-MetStat Panel (Cat # 19-1002k, EpiCypher), which contains 16 different DNA-barcoded histone modifications, was added into the H3K4me3 and IgG samples as spike-in controls before antibody binding. pAG-MNase binding, chromatin digestion and DNA purification were performed following the manufacture’s protocol (User Manual v3.3). 0.5 ng E. coli Spike-in DNA (Cat# 18-1401, EpiCypher) was added to each reaction for downstream data normalization.

Library preparation was performed with the NEBNext® Ultra™ II DNA Library Prep Kit (E7645S, NEB), with PCR amplification parameters as [98 °C for 45 seconds, 13 cycles of (98 °C for 15 seconds, 60 °C for 10 seconds), 72 °C for 60 seconds and 4 °C hold]. NEBNext® Multiplex Oligos (E6440S, NEB) were used for library indexing, with the i7 and i5 index sequences of each library presented in Table 2. The final libraries were visualized and quantified with a TapeStation instrument, with a representative library trace shown in Fig. 2b, which successfully indicates the periodicity of chromatin structures.

**Table 2:** Index sequences of 18 CUT&RUN libraries presented in this study.

Region	Modification	Replicate	i7	i5
Cortex	H3K27me3	Replicate1	TCCACGTT	GGTTGAAC
		Replicate2	AACCAGAG	CTTCGGTT
	H3K4me3	Replicate1	GTCAGTCA	CGGCATTA
		Replicate2	CCTTCCAT	CACGCAAT
	IgG	Replicate1	AGGAACAC	GGAATGTC
		Replicate2	CTTACAGC	TGGTGAAG
Medulla	H3K27me3	Replicate1	CAGTGCTT	TTCCAGGT
		Replicate2	TCCATTGC	TACGGTCT
	H3K4me3	Replicate1	GTCGATTG	AAGACCGT
		Replicate2	ATAACGCC	CAGGTTCA
	IgG	Replicate1	GCCTTAAC	TAGGAGCT
		Replicate2	GGTATAGG	TACTCCAG
Papilla	H3K27me3	Replicate1	GTAAGGTG	CGAATTGC
		Replicate2	CGAGAGAA	GGAAGAGA
	H3K4me3	Replicate1	CGCAACTA	TCGGATTC
		Replicate2	CACAGACT	CTGTACCA
	IgG	Replicate1	TGGAAGCA	GAGAGTAC
		Replicate2	CAATAGCC	TCTACGCA

### Next-generation sequencing

All 6 dual-indexed Hi-C libraries and 18 dual-indexed CUT&RUN libraries were sequenced on a 300-cycle NovaSeq X Plus platform (Illumina). Sequencing depths of all Hi-C and CUT&RUN libraries are summarized in Tables 3 and 4, respectively. Hi-C libraries were sequenced with an average depth of 578 million reads and CUT&RUN libraries were sequenced with an average depth of 19 million reads.

**Table 3:** Sequencing depths of Hi-C libraries presented in this work.

Region	Replicate	Total Reads
Cortex	Replicate1	552,451,162
	Replicate2	573,392,643
Medulla	Replicate1	482,596,289
	Replicate2	623,193,489
Papilla	Replicate1	677,546,608
	Replicate2	559,329,612

**Table 4:** Sequencing depths of CUT&RUN libraries presented in this work.

Region	Modification	Replicate	Total Reads	E. coli Reads (Bowtie2 alignment)
Cortex	H3K27me3	Replicate1	17,939,046	57,656
		Replicate2	15,548,369	39,133
	H3K4me3	Replicate1	29,670,340	165,311
		Replicate2	20,971,242	114,477
	IgG	Replicate1	20,486,985	134,230
		Replicate2	17,107,957	138,626
Medulla	H3K27me3	Replicate1	19,927,691	110,202
		Replicate2	25,289,963	126,381
	H3K4me3	Replicate1	13,429,807	105,156
		Replicate2	26,763,620	363,567
	IgG	Replicate1	16,126,257	105,183
		Replicate2	21,147,369	170,977
Papilla	H3K27me3	Replicate1	24,922,313	152,521
		Replicate2	20,944,175	123,093
	H3K4me3	Replicate1	23,117,763	304,089
		Replicate2	4,119,522	46,513
	IgG	Replicate1	15,885,706	190,892
		Replicate2	13,094,707	138,635

### Hi-C data processing and analysis

Preprocessing of paired-end raw .fastq files of Hi-C sequencing data were performed with Juicer v1.6^30^, including genome alignment, read sorting, duplicate removal and generation of .hic contact matrices, with resolutions of 5 K, 10 K, 25 K, 50 K, 100 K, 250 K, 500 K, 1000 K and 2500 K bases. Human hg19 (primary assembly of GRCh37) was used as the reference genome. The hg19 .fasta file was used to create an Arima Hi-C restriction enzyme site position file with the generate_site_positions.py script provided by Juicer. All other data preprocessing procedures were performed following the Arima-HiC bioinformatics manual (A160600 version 07/19/2021). Only .hic files with mapping quality over 30 (MAPQ >= 30) were used for downstream analysis in this study.

Finding chromatin contact domains was performed with Arrowhead (implemented in Juicer tools v1.6.2) as previously described at a resolution of 5000 bp^31^. This analytical pipeline was first performed on individual replicate .fastq files, and after quality check, was performed again on .fastq files merged from two replicates of the same kidney anatomic region. Data visualization was performed with Juicebox v1.8.8 and WashU Epigenome Browser^32^. Data files generated through hg38 alignment are also available at 10.5281/zenodo.11955175^29^.

### CUT&RUN data processing and analysis

The paired-end raw.fastq files of CUT&RUN sequencing data were first trimmed with TrimGalore v0.6.4_dev (https://github.com/FelixKrueger/TrimGalore). The reads were aligned to the human hg19 (primary assembly of GRCh37) genome or the E. coli genome (E. coli strain K12, MG1655; available at https://support.illumina.com/sequencing/sequencing_software/igenome.html) for .sam file generation with Bowtie2 v2.3.5.1^33^. The number of E. coli reads of each CUT&RUN library is summarized in Table 4. The .sam files post hg19 genome alignment were sorted and indexed with samtools v1.10^34^ to generate sorted.bam files. For each library, bigwig file were subsequently generated with bamCoverage v3.5.4^35^. This analytical pipeline was first performed on individual replicate .fastq files, and after quality check, was performed again on .fastq files merged from two replicates of the same kidney anatomic region. Evaluation of the SNAP-CUTANA™ K-MetStat Panel was performed on H3K4me3 and IgG libraries with scripts provided by the EpiCypher manufacture.

The peak calling step was performed using the Sparse Enrichment Analysis for CUT&RUN (SEACR) workflow developed by Meers *et al*.^36^ Previously sorted .bam files were first converted to .bed files using the bamtobed function of bedtools. Then, the 5′ and 3′ coordinates of the read pairs were selected to generate a new .bed file, which was subsequently converted to .bedgraph format using the genomecov function of bedtools. The scale factor used in this step followed the Epicypher CUTANA manual, which measures the ratio of mapped reads between each histone marker and its corresponding E. coli Spike-in DNA. After generating the .bedgraph files, SEACR was used to call peaks between each histone modification marker and its region-specific IgG control. The called peaks were exported as .peaks.stringent.bed files for downstream analysis using R v4.2.2. R packages circlize^37^ and karyoploteR^38^ were utilized to generate the density and coverage plots of peaks on each chromosome. The R package ChIPseeker was employed for annotating and visualizing the genomic locations of each peak^39,40^. Data files generated through hg38 alignment are also available at 10.5281/zenodo.11955175^29^.

## Data Records

All primary data have been deposited in NCBI’s Gene Expression Omnibus and are available through GEO Series accession number GSE253634^28^, including (1) raw fastq files of Hi-C sequencing data, (2) processed Hi-C contact matrices for all Hi-C libraries, (3) raw fastq files of CUT&RUN sequencing data and (4) processed bigwig files for all CUT&RUN libraries. Supporting data files, including donor clinical information and intermediate data that are not compliant with the GEO requirement, are available at 10.5281/zenodo.11955175^29^.

## Technical Validation

### Hi-C sequencing data evaluation

**Fig. 3:**
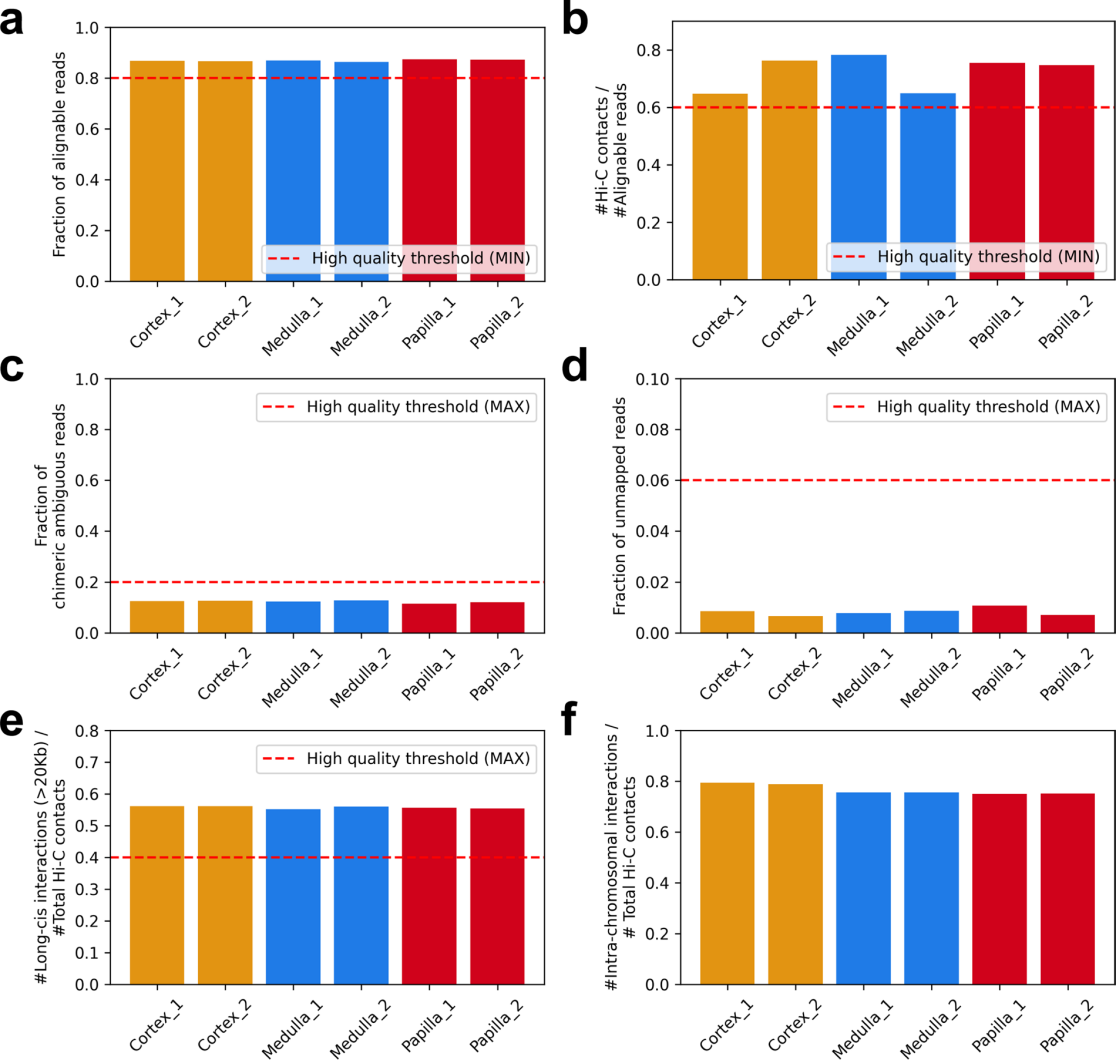
Quality control metrics of Hi-C libraries. Ratios of the alignable read number to the total read number (**a**), the identified Hi-C contact number to the alignable read number (**b**), the chimeric ambiguous read number to the total read number (**c**), the unmapped read number to the total read number (**d**), the long-cis interaction number to the total Hi-C contact number (**e**) and the intra-chromosomal interaction number to the total Hi-C contact number (**f**). Libraries with values above the indicated threshold (a/b/e) or below the indicated threshold (c/d) are considered with high quality.

We analyzed the quality control metrics generated by Juicer^30^ during Hi-C data preprocessing. A total of 687, 797, 625, 675, 269, 740 and 808,998,165 Hi-C contacts were identified in the human kidney cortex, medulla and papilla, respectively. As indicated by the Arima Hi-C manual, high-quality Hi-C libraries are typically characterized by a ratio of alignable reads to total reads over 80% and a ratio of Hi-C contact reads to alignable reads over 60%, and we found the values of all 6 libraries generated in this work are above these thresholds (Fig. 3a,b). Our library quality was also validated by fraction of chimeric ambiguous reads and fraction of unmapped reads lower than their corresponding thresholds for all libraries (Fig. 3c,d).

Since the Hi-C technology mostly captures long-range 3D interactions within chromosomes^18^, we examined ratios of long-cis interactions with a distance greater than 20kb to total Hi-C contact numbers, and identified that for all 6 libraries, over half of the Hi-C contacts present long-cis interactions, with ratios higher than the quality threshold (Fig. 3e). Over 70% of Hi-C contacts are enriched for intra-chromosomal interactions for all libraries (Fig. 3f), consistent with expectation.

### Evaluation of Hi-C contact matrices

**Fig. 4:**
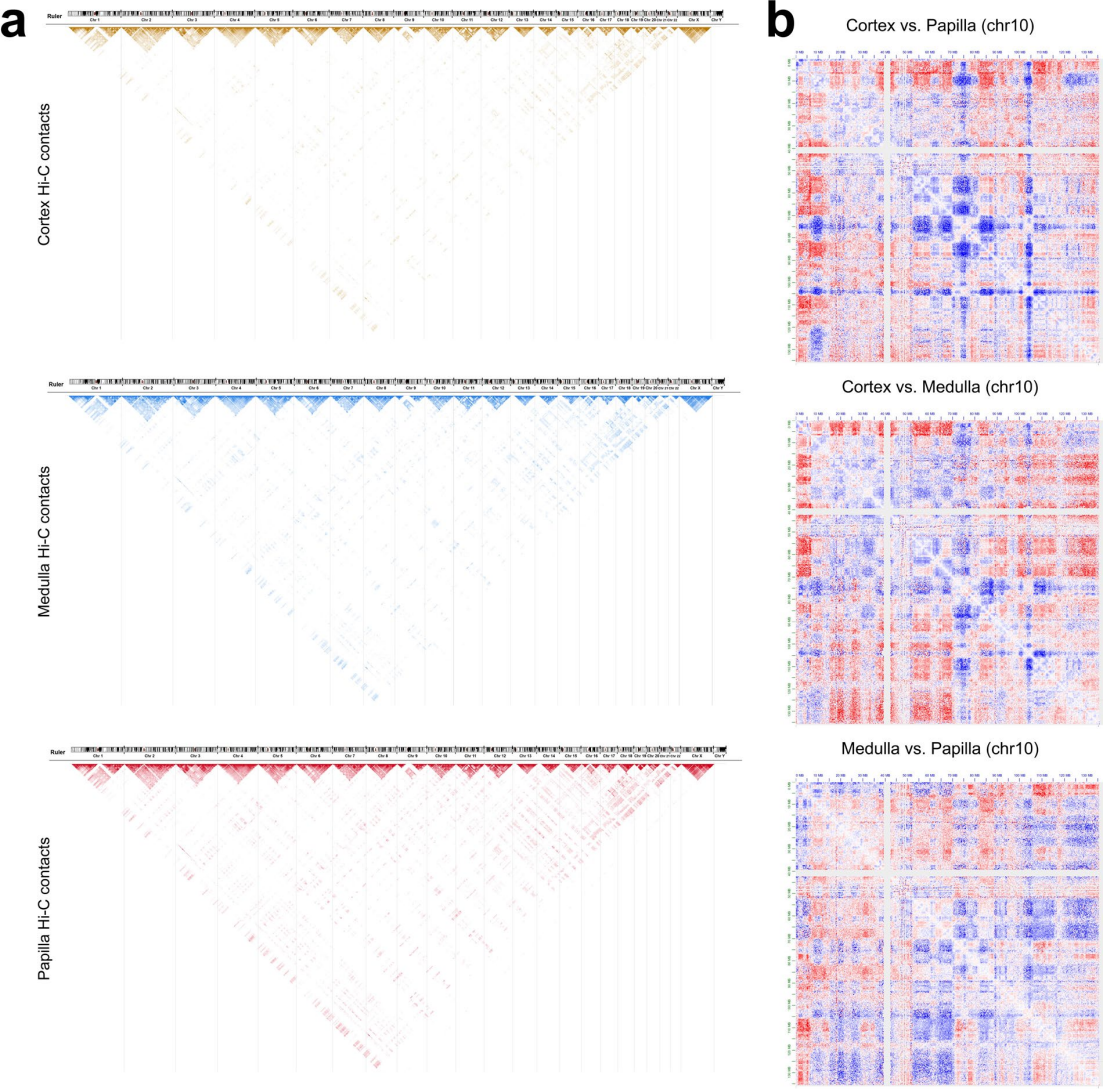
Hi-C contact matrix analysis across the genome. (**a**) Visualization of Hi-C contact matrices of human kidney cortex, medulla and papilla across all chromosomes. Maximum contact score is set as 10,000 and contacts with score lower than 500 are filtered for the convenience of data visualization. (**b**) Visualization of differences of Hi-C contact matrices between kidney cortex and papilla (top), cortex and medulla (middle) and medulla and papilla (bottom) in chromosome 10. The red color indicates a positive value and the blue color indicates a negative value after subtraction. Matrix files generated from merged duplicates are used.

We projected all Hi-C contacts onto the hg19 genome for all kidney anatomic regions. As mentioned above, most Hi-C contacts are enriched for intra-chromosomal interactions (Fig. 4a) for all autosomes and the X chromosome. The Hi-C contact maps of kidney cortex, medulla and papilla share a similar pattern when we examined them across all chromosomes (Fig. 4a), as a result of conserved chromatin conformation in mammalian cells. On the other hand, distinct Hi-C contact profiles can be observed when examined within a chromosome or specific genomic regions (Fig. 4b), indicating different chromatin interaction networks across kidney anatomic regions.

**Fig. 5:**
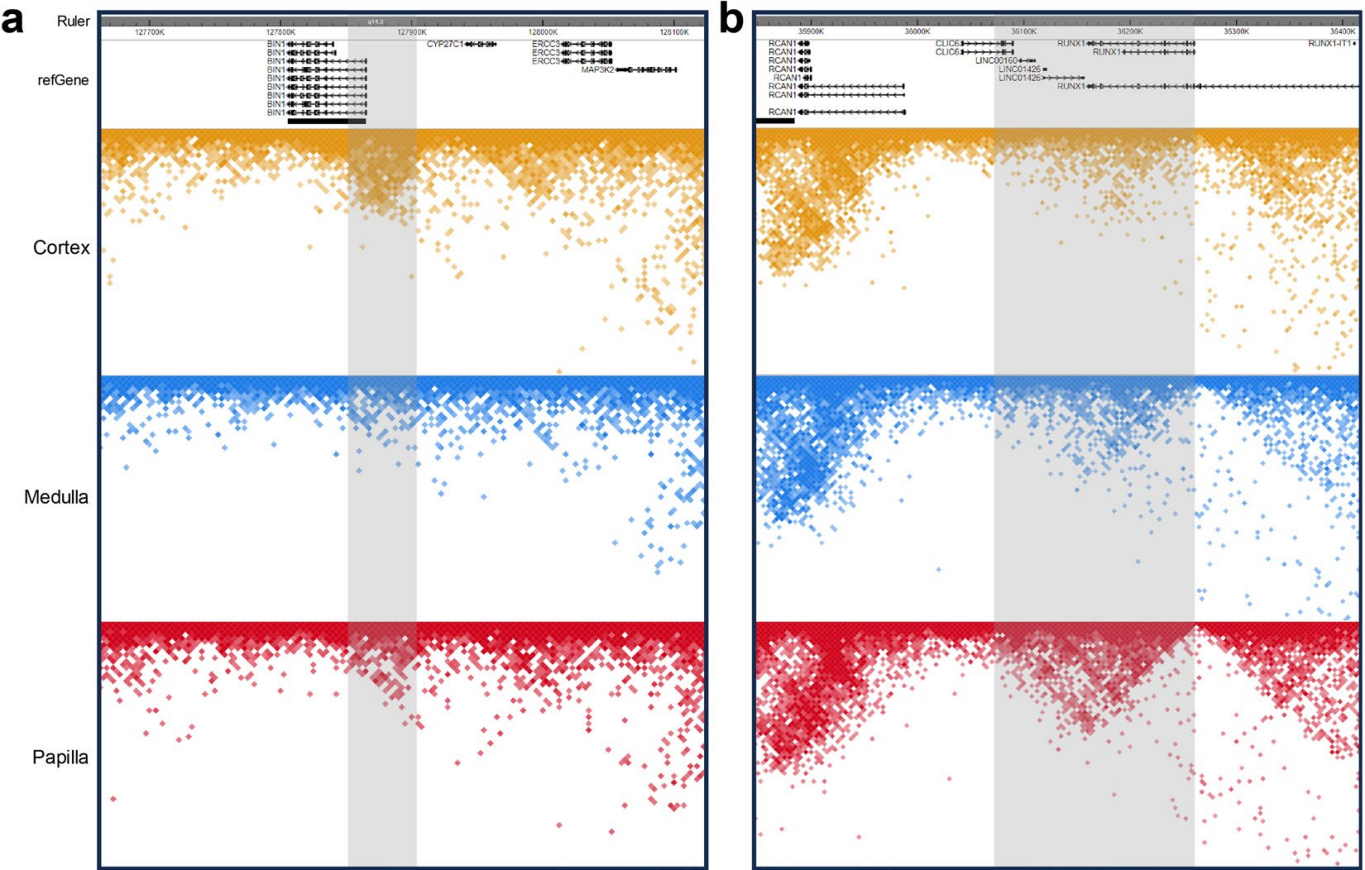
Visualization of chromatin interactions for specific genomic loci. (**a**) Increased Hi-C contacts in kidney cortex than medulla and papilla, nearby *BIN1* promoter region. (**b**) Increased Hi-C contacts in kidney medulla and papilla than cortex in a *RUNX1*-containing region. Data are visualized at a resolution of 5,000 bp and Square-Root Vanilla-Coverage normalization is used for visualization.

For example, chromatin contact domain calling analysis with Arrowhead^31^ identified a domain located nearby *BIN1* promoter region in the kidney cortex, but not in kidney medulla and papilla, and we observed more Hi-C contacts within this region in the kidney cortex (Fig. 5a). This observation is concordant with the identification of high *BIN1* gene expression in proximal tubular cells in recent single-cell studies^6,41,42^. We recently described increased *RUNX1* gene expression and chromatin accessibility along the human kidney corticopapillary axis^16^, and consistently, our Hi-C sequencing data revealed a domain covering *RUNX1* with more chromatin interactions in medulla and papilla than that in the kidney cortex (Fig. 5b).

### CUT&RUN sequencing data evaluation

**Fig. 6:**
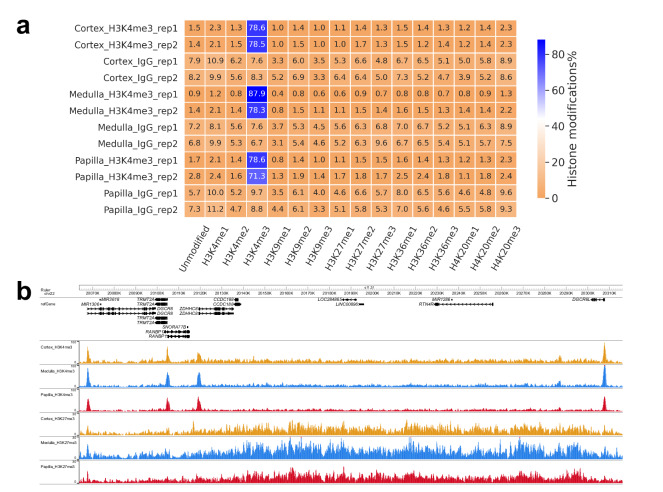
CUT&RUN data evaluation. (**a**) Heatmap showing percentage of reads mapping to each histone modification in the K-MetStat Panel in the H3K4me3 and IgG libraries. (**b**) Visualization of CUT&RUN signals on a selected genomic region. Bigwig files generated from merged duplicates are used.

For all 16 CUT&RUN libraries, an average of 97.8% of the total reads (from 97.09% to 98.62%) were mapped to the hg19 genome. As mentioned in Methods, E. coli Spike-in DNA was added to each library (Table 4) and we found an average of 0.8% of the total reads (from 0.26% to 1.39%) were mapped to the E. coli genome during Bowtie2 alignment, consistent with expectation. For H3K4me3 and IgG libraries in which the SNAP-CUTANA K-MetStat Panel was added as spike-in controls (see Methods), the ratio of reads containing exogenous histone modification barcodes to the total reads is consistently <0.6%. Analyzing the composition of the K-MetStat Panel revealed an enrichment for H3K4me3 barcodes in the H3K4me3 CUT&RUN libraries, indicating a strong on-target effect (Fig. 6a). Since H3K4me3 typically marks the transcription start sites (TSSs) and indicates active transcription, and H3K27me3 is a regressive histone marker^20,43–45^, we examined CUT&RUN signals of the two histone modification markers, and identified sharp H3K4me3 peaks localized to TSSs at gene promoter regions, as well as broad H3K27me3 peaks over repressive genomic regions, suggesting successful chromatin modification profiling (Fig. 6b).

### CUT&RUN data peak calling evaluation

**Fig. 7:**
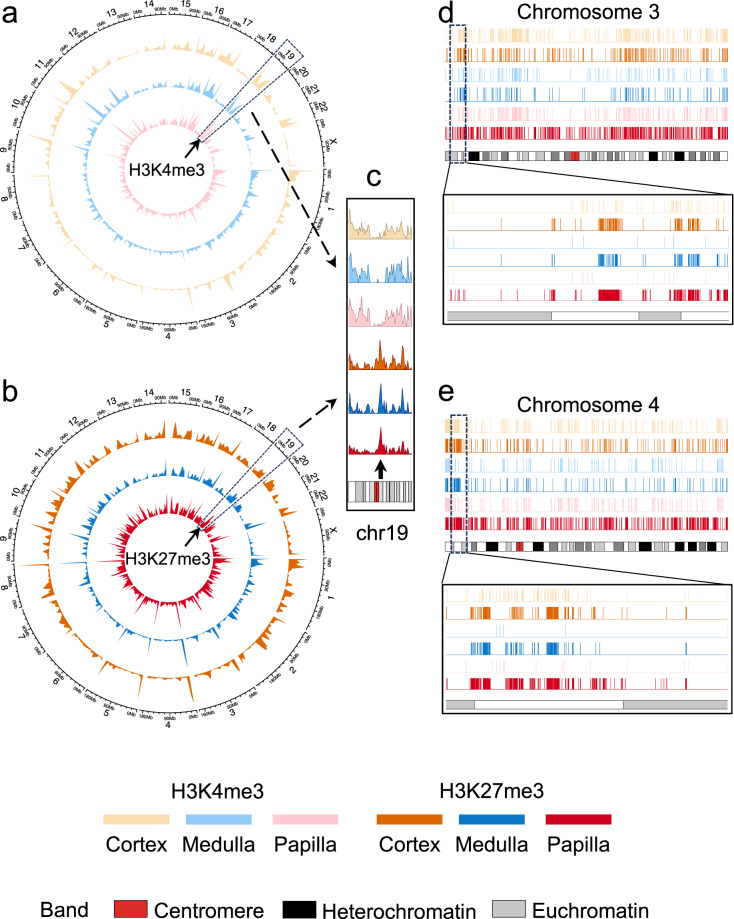
Visualization of CUT&RUN peaks. (**a**,**b**) Circos plots for genome-wide H3K4me3 (**a**) and H3K27me3 (**b**) peak profiles across kidney cortex (outer layer), medulla (middle layer) and papilla (inner layer). Sizes of each chromosome are shown. A region of interest on chromosome 19 is highlighted with arrows and is shown with zoom-in (**c**). (**d**,**e**) Coverage plots of the called peaks over chromosome 3 (**d**) and chromosome 4 (**e**) for each CUT&RUN library (with duplicates merged).

To evaluate the efficacy of our CUT&RUN dataset in downstream analysis, we performed peak calling analysis to identify genome-wide CUT&RUN signal-enriched regions for each library with a computational pipeline previously described^36^, where the IgG CUT&RUN profile of each kidney region was subtracted as a negative control to eliminate background noise. A total of 8726, 2153 and 4955 H3K4me3 peaks, and a total of 10864, 5125 and 24184 H3K27me3 peaks, were identified in the human kidney cortex, medulla and papilla, respectively. Many genomic regions show different relative abundances of H3K4me3 and H3K27me3 peaks. For example, a chromosome 19 region exhibits low presence of H3K4me3 peaks while high abundance of H3K27me3 peaks across the three kidney anatomic regions (Fig. 7a,b; highlighted with arrows; Fig. 7c for a zoom-in view). Differences in peak distribution across three kidney regions are evident, with coverage plots of representative chromosomes shown in Fig. 7d,e. As another example, for kidney cortical genome-wide H3K4me3 peaks, there are 97.3% of medullary H3K4me3 peaks exhibiting genomic overlaps with them, while only 16.0% of cortical H3K27me3 peaks coincide with them, supporting existing knowledge that the two histone modifications typically mark distinct genomic regions.

**Fig. 8:**
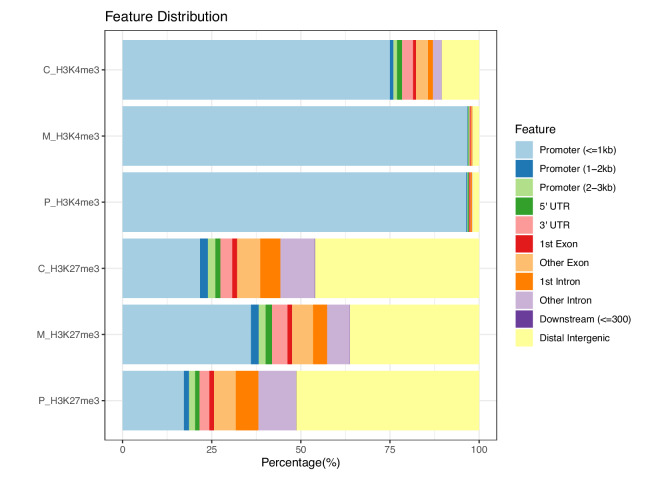
Genomic location annotations of CUT&RUN peaks. Distribution of genomic locations of the called peaks for each library (with duplicates merged). Annotations was provided by ChIPseeker^39,40^.

Furthermore, annotation of genomic locations for the called peaks revealed that over 75% of the H3K4me3 peaks reside within the promoter regions, being less than or equal to 1 kb from the TSSs, for all three kidney regions (Fig. 8). Conversely, 34%–51% of the H3K27me3 peaks are located in distal intergenic regions, indicative of a preference for a cis-regulatory role. These observations are consistent with previous reports^46,47^.

## Data Availability

Scripts for Hi-C sequencing data preprocessing, CUT&RUN sequencing data preprocessing and analysis relevant to figures in this study are available at https://github.com/TheHumphreysLab/kidney_anatomy_chromatin. Versions of all required packages are described in the scripts and Methods.
